# Chromosome-scale genome assembly and annotation of two geographically distinct strains of malaria vector *Anopheles albimanus*

**DOI:** 10.1038/s41598-025-01713-9

**Published:** 2025-06-03

**Authors:** Dieunel Derilus, Gareth D. Weedall, Michael W. Vandewege, Dhwani Batra, Mili Sheth, Lori A. Rowe, Ananias A. Escalante, Audrey Lenhart, Lucy Mackenzie Impoinvil

**Affiliations:** 1 Entomology Branch, Division of Parasitic Diseases and Malaria, National Center for Emerging and Zoonotic Infectious Diseases, Centers for Disease Control and Prevention, Atlanta, GA USA; 2 School of Biological and Environmental Sciences, Liverpool John Moores University, Liverpool, UK; 3 Department of Clinical Sciences, College of Veterinary Medicine, North Carolina State University, Raleigh, NC 27607 USA; 4 Office of Advanced Molecular Detection, Division of Infectious Disease Readiness and Innovation, Centers for Disease Control and Prevention, Atlanta, GA USA; 5 Biotechnology Core Facility Branch, Division of Core Laboratory Services and Response. Office of Laboratory Safety and Response. Centers for Disease Control and Prevention, Atlanta, GA USA; 6 Department of Microbiology, Viral Characterization, Isolation, Production and Sequencing Core, Tulane National Primate Research Center, Tulane University, Covington, LA USA; 7 Department of Biology/Institute for Genomics and Evolutionary Medicine, Temple University, Philadelphia, PA USA

**Keywords:** Hybrid assembly, *Anopheles albimanus*, Stecla, Cartagena, Mosquito genome, PacBio Sequencing, Illumina Sequencing, Comparative genomics, Structural variation

## Abstract

*Anopheles albimanus* is one of the principal malaria vectors in the Americas and exhibits phenotypic variation across its geographic distribution. High-quality reference genomes from geographically distant populations are essential to deepen our understanding of the biology, evolution, and genetic variation of this important malaria vector**.** In this study, we applied long-read PacBio and short-read Illumina sequencing technologies to assemble the complete genomes of two reference strains of *An. albimanus,* Stecla (originating from El Salvador), and Cartagena (originating from Colombia); and investigated the structural features of these genomes, including gene content, transposable elements (TEs), genetic variation, and structural rearrangements. Our hybrid assembly approach generated reference-quality genomes for each strain and recovered ~ 96% of the expected genome size. The genome assemblies of Stecla and Cartagena consisted of 109 and 149 scaffolds, with estimated genome sizes of 167.5 Mbp (N_50_ = 88 Mbp) and 167.1 Mbp (N_50_ = 87 Mbp), respectively. They exhibited a high level of completeness and contained a smaller number of gaps and ambiguous bases than either of the two previously published reference genomes for this species, suggesting a considerable improvement in the quality and completeness of the assemblies. A total of 12,082 and 12,120 protein-coding genes were predicted in Stecla and Cartagena, respectively. TE analyses indicated more repetitive content was captured in the long read assemblies. The assembled genomes shared 98.12% pairwise identity and synteny analyses suggested that gene position was conserved between both strains. These newly assembled genomes will serve as an important resource for future research in comparative genomics, proteomics, epigenetics, transcriptomics, and functional analysis of this important malaria vector.

## Introduction

*Anopheles albimanus* belongs to the subgenus Nyssorhynchus of which the majority of species are widely distributed in the neotropics, with the exception of *An. albimanus* which extends to the Nearctic region^1^. It is an important contributor to malaria transmission primarily in coastal areas throughout the Caribbean region, Central and South America^1–5^. *An. albimanus* exhibits phenotypic variation including morphological variations in the larval stages^6^, and occurs in a range of diverse habitats^1,7^. It has also been found naturally infected with *Plasmodium* in nearly every country in which it is encountered^8^. Populations can differ widely in their host preference and vectorial capacity, and have been reported as anthropophilic, zoophilic, exophagic, and generally exophilic in host-seeking and feeding behavior^1,9^. This heterogeneity in key behavioral, ecological, and environmental factors may reflect intraspecific differentiation. Cryptic species is common in the *Anopheles* genus, particularly within the *An. gambiae* complex, where nine cryptic species have been identified^10–12^. Although there is no evidence of cryptic species within *An. albimanus*, the possibility remains open due to its high phenotypic variation.

Extensive research has been done on *An. albimanus* ecology^3,7,13,14^, vector competence^15^, evolution^16^, insecticide resistance^17–19^, and feeding behavior^20–22^. However, limited efforts have been made to generate high-quality genome assemblies of different strains of this species^16,23,24^. Due to its wide geographical range and habitat distribution, the genetic diversity of *An. albimanus* cannot be captured with the genome assembly of a single strain of this species. To date, the three main efforts to generate genome sequence assemblies of this species are AalbS2^24^, the recently published COL albi^16^, and AalbS3^23^. However, these three assemblies were generated from only the *An. albimanus* Stecla strain. Additionally, the first two assemblies (AalbS2 and albi) were highly fragmented and featured many gaps, likely because they were generated using only Illumina short read sequencing. The third assembly (AalbS3) was generated using a combination of long-read Oxford Nanopore sequencing, Illumina short read sequencing, Illumina, Hi-C, and optical mapping, which significantly improved the contiguity and completeness of the assembly^23^.

The main limitations of short-read sequencing are the inability to fully capture the entire length of transcripts in eukaryotic genomes, as well as the potential for PCR amplification bias during library construction^25,26^. In contrast, long-read sequencing improves de novo assembly, mapping accuracy, and helps in identifying transcript isoforms and the detection of structural variants. Additionally, sequencing of native DNA/RNA molecules with long reads removes amplification bias and preserves base modifications^27^. Combining short but accurate Illumina reads (< 1% error rate) with longer but less accurate reads from Pacific Biosciences (PacBio) or Oxford Nanopore, can generate more contiguous, accurate, and complete genome assemblies than Illumina alone^28,29^. Importantly, the two long-read sequencing approaches are known to differ in accuracy and in the sequencing chemistry. The PacBio sequencer reads molecules multiple times (~ 10 times in average) to generate high-quality continuous long reads, while Oxford Nanopore can only sequence a molecule twice^30^^30^.. The raw base-called error rate for PacBio has improved in recent years to < 1% in circular consensus reads (CSS)^31^ and ~ 5% for ONT sequences^32^. Consequently, a hybrid assembly generated with data from both Illumina and PacBio is expected to be more accurate and of higher resolution than those co-assembled with Illumina and Oxford Nanopore. However, no *An. albimanus* genome has previously been co-assembled with Illumina and PacBio data, nor has a high-quality genome assembly been generated for the Cartagena strain of *An. albimanus* (originating from Colombia) and compared with the Stecla strain (originating from El Salvador).

Here we present the hybrid assemblies of two strains of *An. albimanus* (Stecla and Cartagena), generated by combining Illumina short-read and PacBio long-read sequencing. The annotation of the assembled genomes was supported by RNA-Seq data, improving exon–intron structure prediction. The resulting assemblies exhibit high levels of completeness, contiguity and accuracy, outperforming previous non-hybrid and hybrid assemblies of *An. albimanus*. This study presents the first genome assembly of the Cartagena strain, and the first hybrid assembly of the Stecla strain co-assembled using PacBio and Illumina data. Gene content, single nucleotide polymorphisms (SNPs), Transposable Elements (TEs) and several other genomic features were characterized and discussed. These assemblies provide a useful resource for comparative genomics, proteomics, epigenetics, transcriptomics, and functional analysis of *An. albimanus*, and will contribute to our understanding of the biology and the evolution of this important malaria vector.

## Materials and methods

### Mosquito rearing and DNA preparation

Two reference strains of insecticide susceptible *An. albimanus* were reared from established laboratory colonies. The Stecla strain (hereafter named “STEC”), originally colonized from El Salvador, and the Cartagena stain (hereafter named “CART”), originally colonized from Colombia, were reared in the insectary at the U.S. Centers for Disease Control and Prevention (CDC), Atlanta, Georgia, USA. Mosquitoes were maintained at a constant 27 ± 2 °C and 70 ± 10% humidity on a 14:10 h light:dark cycle and adults were provided 10% sucrose ad libitum. Three to five-day-old adult female mosquitoes were obtained from isofemale lines established from a single mated female mosquito from each colony. Thirty-five females were obtained from the STEC colony and 54 from the CART colony. Mosquitoes were killed by freezing and stored at −80 °C until DNA extraction.

DNA was extracted from the pools of mosquitoes (35 mosquitoes for STEC and 54 for CART) using the Qiagen genomic-tip 500/G (Qiagen, Valencia, CA) to generate high molecular weight genomic DNA. DNA concentration was assessed and quantified using the NanoDrop 2000 spectrophotometer (Thermo Scientific ™ NanoDrop™ 2000 spectrophotometer). The DNA extraction yielded 208.5 and 125.7 ng/µl for CART and STEC, respectively. Equimolar amounts were used for Pacific Biosciences (PacBio) and Illumina HiSeq sequencing according to the manufacturer’s instructions.

### Pacific Biosciences library construction, sequencing, and assembly

Genomic DNA was sheared to 20-kb using needle shearing. Libraries were generated with the SMRTbell Template Prep Kit 1.0 (Pacific Biosciences, Menlo Park, CA) following the standard Pacific Biosciences protocol. The libraries were then size selected on a Blue Pippin (Sage Science, Beverly, MA) with a cutoff size of 10 kb. Libraries were bound to polymerase using the DNA/Polymerase Binding Kit P6v2 (Pacific Biosciences, Menlo Park, CA) and were loaded on 9 SMRTcells (Pacific Biosciences, Menlo Park, CA) and sequenced with C4v2 chemistry (Pacific Biosciences, Menlo Park, CA) for 360 min movies on the RSII instrument (Pacific Biosciences, Menlo Park, CA). Nine SMRT cells were sequenced, generating three *bax.5* files containing the base calling information. These were converted to fastq format using pbh5 tools package from Pacific Biosciences (www.pacbiodevnet.com) and concatenated into one fastq file, which was used as input for the assemblers. The sample processing workflow and hybrid assembly pipeline used to integrate the Illumina short reads and the PacBio long reads are illustrated in **Figure S1.**

### Illumina HiSeq library preparation

Genomic DNA was sheared to a mean size of 600 bp using a Covaris LE220 focused ultrasonicator (Covaris Inc., Woburn, MA) and cleaned using AMpure beads (Beckman Coulter Inc., Indianapolis, IN). The fragmented DNA was utilized to generate dual-indexed sequencing libraries using NEBNext Ultra library prep reagents (New England Biolabs Inc., Ipswich, MA) and barcoding indices synthesized in the CDC Biotechnology Core Facility. Libraries were analyzed for size and concentration, then normalized and pooled. The final pool was subsequently diluted and denatured for loading onto flow cells for cluster generation. Sequencing was performed on an Illumina HiSeq 2500 high output mode using 2 × 125 bp parameters. On completion, base calling, demultiplexing and quality filtering were carried out using bcl2fastq (v2.19.1, Illumina).

### Genome assembly

#### Hybrid assembly the PacBio long reads and Illumina short reads

Prior to hybrid assembly, adapters were trimmed, and low quality reads were removed from the Illumina short reads using the default parameters of fastp (v0.20) software^33^. The genome sizes of the two strains of *An albimanus* were estimated from Illumina short reads data based on* k*-mer distribution using Jellyfish (v2.3.1)^34^ in conjunction with GenomeScope (v1.0.0)^35^. This analysis estimated a genome size of 162.6 Mb and 162 Mb for CART and STEC, respectively. The hybrid assembly of each strain was generated using MaSuRCA assembler (v3.3.4)^29^ as follows. The configuration file was edited by adding our raw datasets with those following non-default parameters: FLYE_ASSEMBLY = 1, JF_SIZE = 1,730,000,000 (10 × the estimated genome size as recommended in the manual), and NUM_THREADS = 28. The expected genome size of 173 Mb was based on the recent hybrid genome assembly of *An. albimanus* (AalbS3)^24^, as it was larger than the estimate from k-mer distribution. A shell script was then generated from the configuration file, which was executed to assemble the raw sequence data. Using those parameters, the preliminary raw assembly was generated following three main steps: 1) transforming the Illumina paired-end reads into ‘super-reads’, which are best suited for correcting the long read due to the longer length and lower coverage; 2) reconstructing the long and accurate ‘mega-reads’, by computing the approximate alignment of all resulted ‘super-reads’ to the PacBio long reads; 3) assembling the ‘mega-reads’ into contigs and scaffolds using Flye^36^, which is supplied and installed with MaSuRCA.

### Removing chimeric and contaminated contigs

A BLASTx search of the resulting contigs/scaffolds for each assembly was performed against the NCBI nr database (2022) to filter out chimeric contigs, low-complexity sequence and potential contamination. Contigs whose best matches were not assigned to Arthropoda, (tagged as probable contamination or low-complexity DNA) were removed. The remaining contigs were polished with the Illumina paired reads corresponding to each strain using POLCA (included in MaSuRCA, which is supplied and installed with MaSuRCA^29^^37^,

### Chromosome scale scaffolding of pre-assembled contigs

To assign contigs to chromosomes, we used the chromosome scaffolding script included in MaSuRCA (chromosome_scaffolder.sh), with default parameter^29^. We provided the reference genome AalbS3 as input^23^ (-r option), the draft assembly to be scaffolded (-q option) and the raw PacBio long reads (-s option) as parameters. This scaffolding script identified mis-assemblies in pre-polished contigs using the reference alignments and ordered the cleaned into chromosomes. This program generated a chromosome level assembly (with some unplaced contigs) for each strain, which was subsequently re-polished using POLCA (as described above)^29^^37^,, followed by gap-filling with the PacBio long reads and the Illumina short reads using TGS-GapCloser (v1.0.1)^38^. This gap-filling step was added to our pipeline because POLCA polishing does not fill gaps but fixes only SNPs and small indels. Lastly, the resulting assembly for each strain was concatenated with their respective mitogenome, and repetitive DNA was soft masked using RepeatMasker (v4.0.8)^39^. The whole hybrid genome assembly generated was used for downstream analysis. The level of similarity and synteny relationship between the two assemblies was performed using the nucmer utility of MUMmer (v4)^40^

### Assessment of genome assembly

The quality of the assembled genomes was evaluated using four methods. First, the basic statistical data for each assembled genome, including the number of contigs, N50 contig lengths, number of gaps, and ambiguous bases (Ns) were computed using QUAST(v5.0.2)^41^ and compared with the two previously published genomes of this species. Second, the trimmed and short PE reads used for PacBio long read correction were mapped to the assemblies with Bowtie2 (v2.3.5)^42^, and the mapping rate was computed using Samtools (v1.9)^43^. Third, ~ 18 Gb of RNA-Seq Illumina PE reads previously generated for *An. albimanus* were mapped to the genome assemblies using ‘subjunc’ (v2.0.1)^44^, followed by alignment sorting and filtering using Samtools (v1.9)^43^ as previously described^17,45^. Fourth, Benchmarking Universal Single-Copy Orthologs (BUSCO:v5)^46^ was used to evaluate the quality and the completeness of each genome assembly. For comparison, all four genome assembly assessment methods were also performed on two previously published genomes of the species: AalbS2^24^ and AalbS3^47^.

### Identifying the mitochondrial genome

The complete 15–16 kb sequences of published mitochondrial (mt) genomes of 5 *Anopheles* species including *An. darlingi* (NC_014275.1), *An. funestus* (MT917167.1), *An. gambiae* (NC_002084.1), *An. sacharovi* (MW366634.1) and *An. sinensis (*MG816549.1) were downloaded from NCBI. These were BLASTx-searched against the de novo assembly of each *An. albimanus* strain. Three contigs produced significant alignments with the query mt genomes for STEC (88–93% id) and CART (83–96% id), respectively. From the alignment generated by the blast results, we recovered the mt genomes by carefully breaking each contig fragment successfully assigned to the reference mitogenome sequences and joining them accordingly. The resulting mitogenomes were further gap-filled and replaced in the draft assembly for downstream analysis. The annotation of the mt genomes was performed using MITOZ (v3.5)^48^ and visualized using MacVector (https://macvector.com).

### Gene prediction and functional annotation

Gene prediction of the soft-masked assemblies was performed using Braker2 (v2.1.6) pipelines using RNA-Seq and protein data as recommended in the manual^49^.Three RNA-Seq replicate libraries previously generated from STEC colonies of *An. albimanus* (NCBI accession numbers: SRR8128634, SRR8128636, SRR8128637)^17^ were individually mapped to the soft-masked genomes using ‘subjunc’, part of the subread aligner (v2.0.1)^44^, with default parameters. The resulting alignment was filtered to remove reads with low mapping quality (q < 10) and sorted using Samtools^43^ to generate three BAM files. Protein families corresponding to all species belonging to the *Arthropoda* lineage were extracted from OrthodDB protein database (https://v100.orthodb.org/download/odb10_arthropoda_fasta.tar.gz) and concatenated in a single protein file. The gene predictions based on protein homology and RNA-Seq alignment were performed separately using the *braker.pl* using ‘-softmasking’ option. The two gene prediction results (braker.gtf) were combined using TSEBRA (Transcript Selector for BRAKER)^50^. Finally, the predicted-protein coding genes, CDS, and transcripts were extracted from each genome assembly with their corresponding TSEBRA output (gtf), using gffread (v0.12.1)^51^.

Predicted genes were functionally annotated using Blast2GO as follows. First, a local BLASTp (v2.9) search of the predicted protein coding sequences was conducted against the Arthropoda (taxid = 6665) category of the nr protein NCBI database with maximum e-value cut-off 10^–3^. Second, the protein sequences were searched against the InterPro database^52^ using InterProScan (v5)^53^. The Blastp and InterProscan outputs were simultaneously provided to the Blast2GO command line, which mapped the RefSeq and InteProScan identifiers to the GO database as curated and updated in the last release of the Blast2GO database (July 2021).We used MCscan python-version (v1.1.18) with default parameters to inspect synteny conservation between the STEC and CART strains and identify potential duplications or inversions^54^. The longest transcript was selected to represent each gene, and Mcscan used LASTAL as the default sequence alignment tool^55^.

### Genome-wide analysis of single nucleotide polymorphisms

To estimate the genome-wide genetic diversity and differences between the two strains, the cleaned Illumina PE short reads from both STEC and CART were mapped to the newly assembled STEC reference genome using Bowtie2 (Langmead & Salzberg, 2012). Duplicate reads were removed using Picard tool (https://broadinstitute.github.io/picard), SNP calling was performed using SNver^56^, and SNPs were annotated using SnpEff (v4.3)^57^

### Transposable elements analysis

To estimate repetitive DNA content, assemblies of STEC, CART, and AalbS2 were concatenated together and searched with RepeatModeler^58^ following the protocol in Platt et al*.*^59^. Briefly, genomes were searched de novo for repeats using RepeatModeler. To curate and improve the RepeatModeler output, the resulting consensus sequences were BLASTed against the three genomes. For each element, the best 50 hits separated by at least 2,000 bp (to avoid tandem elements), plus 1000 bp of the flanking sequence were extracted and aligned using MAFFT^60^. From each alignment, we reconstructed a majority rule consensus sequence. This process was repeated until single copy DNA was identifiable on the 5’ and 3’ ends of the alignment. We used BLAST to identify elements that were more than 95% similar. In those cases, the longest element was chosen to represent the TE family. We used RepBase’s CENSOR^61^ and TE class^62^ to classify elements as DNA transposons, Long INterspersed Elements (LINEs), Short INterspersed Elements (SINEs) and Long Terminal Repeats (LTRs). When there was incongruence between identification methods, we viewed alignments for diagnostic structures (LTRs, TIRs, poly-A tails, etc.); if features could not be identified, the element was labelled as unclassified. To understand the repeat content of the AalbS2, AalbS3 and our assemblies, the resulting library of consensus sequences was used to query each genome using RepeatMasker^58^.

## Results and discussion

### Illumina and PacBio sequencing

We used the combination of Illumina short-read and PacBio long-read sequencing to construct the hybrid genome assemblies of two strains of *Anopheles albimanus*: STEC (originating from El Salvador) and CART (originating from Colombia). Illumina sequencing generated a total of 462,584,846 (STEC) and 403,726,572 (CART) paired-end reads, representing 58.2 Gb and 50.8 Gb of sequences for an estimated fold-coverage of 336 × and 294 × for STEC and CART, respectively (assuming a genome size of 173 Mb as reported by^23^). PacBio sequencing generated a total of 1,675,245 (STEC) and 1,444,111 (CART) long reads, representing 8.6 Gb and 10.0 Gb of sequence, or an estimated fold-coverage of 50 × and 58 × for STEC and CART, respectively (**Table S1**). Together, the raw data generated by the two sequencing approaches had sufficiently good coverage to produce good contiguous assemblies of the genomes, as the MaSuRCA software required a minimum of 100 × coverage from paired-end Illumina short reads combined with 10 × coverage from PacBio long reads to generate a good quality assembly^29^.

### Genome assembly

**Fig. 1:**
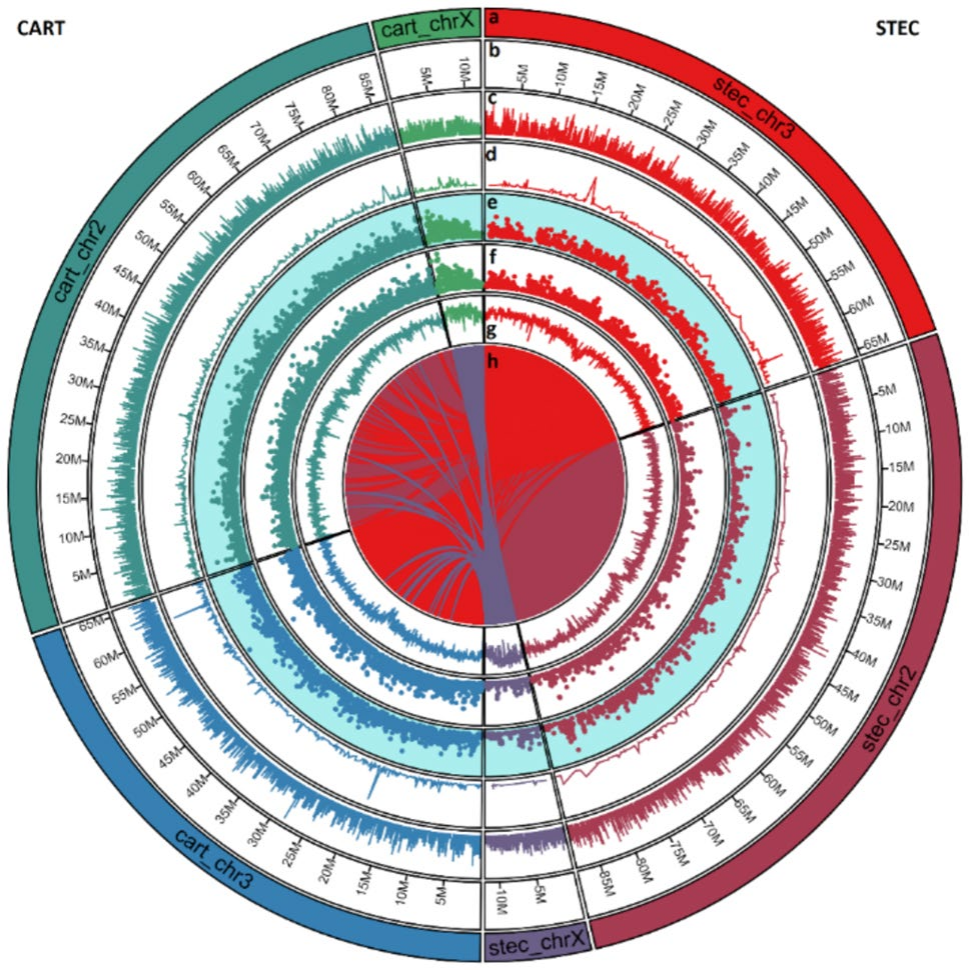
Chromosomal features of the genome assemblies of *An. albimanus strains* CART (left half) and STEC (right half). The concentric circles show from outside to inside: the chromosome name (**a**) and size (**b**), gene density (**c**), non-synonymous SNPs (**d**), insertions (**e**), deletions (**f**), GC content (**g**) and ribbons connecting the two genomes link-up homologous DNA segments between the two assemblies with STEC as reference (**h**). All these genomic features, apart from the last one (**g**) are shown in 10,000 bp sliding windows with a 5,000 bp step size. The three chromosomes of each strain are represented in different colors, and the ribbons (connections) are color-coded to correspond to the three chromosomes of STEC used as the reference for the alignment. The Circo plot was generated Circa (https://omgenomics.com/circa/).

The de novo hybrid assembly (long PacBio reads + short Illumina reads) and non-hybrid assembly (PacBio long reads only) were performed using MaSuRCA and Flye, respectively, followed by error correction and bacterial contig removal to produce high quality assemblies of the two *An. albimanus* strains. The summary statistics of the de novo assemblies (before chromosome scaffolding) are shown in **Table S2**. These de novo assemblies were scaffolded against the AalbS3 reference genome^23^, gap-filled using the Illumina short reads, and error-corrected (see Methods). As expected, the hybrid assemblies exhibited higher contiguity and accuracy than their non-hybrid counterparts for both strains (**Table S3**). The higher quality of the hybrid assemblies is evidenced by their higher contiguity, and fewer errors and gaps relative to the non-hybrid assembly generated with Flye. For the STEC strain, the hybrid assembly generated with MaSuRCA contained 109 scaffolds of 167.4 Mb total length (N50 = 88.2 Mb), with 60 gap regions, and 130 N’s per 100kbp, while the non-hybrid assembly generated with Flye contained 267 contigs of total length 169.3 Mb (N50 = 88.7 Mb), with 127 gap regions and 256 N’s per 100 kbp. For the CART strain, the hybrid assembly contained 149 contigs of 167.06 Mb total length, with 23 gap regions and 76 N’s per 100 kbp was generated, while the non-hybrid assembly contained 184 contigs of 168.3 Mb total length (N50 = 88.3), with 43 gap regions and 145 N’s per 100 kbp (**Table S3, **Fig. 1).

Based on comparison of the results of all the assemblies, we decided to proceed with the hybrid assembly generated with MaSuRCA for further steps in the assembly pipeline, since they exhibited higher contiguity and accuracy, enabling a more accurate and robust downstream analysis. Finally, each assembly consisted of 3 chromosome-level scaffolds (X, 2, and3), the complete mitochondrial (mt) genome, and several unplaced scaffolds (Fig. 1). The genome size of each final assembly was ~ 167 Mb with an average GC content of 49% and represented 97% of the expected genome size (173 Mb). The summary statistics of the two hybrid assemblies generated in this study as compared with two previously published assemblies of *An. albimanus* (AalbS2 and AalbS3) are reported in Table 1, while the distribution of several genomic features such as gene density, non-synonymous SNPs, insertions, deletions, and GC content across the three assembled chromosomes for each strain is depicted in Fig. 1

**Table 1:** Comparison of basic statistics and BUSCO assessment results of the hybrid genome assemblies and two other previously assembled genomes (AlbS2 and AalbS3) of the same species.

Reference	This study		Other published genomes	
Quality metrics	**STEC**	**CART**	**AlbS2**^24^	**AalbS3**^23^
Number of scaffolds	109	149	201	7
Longest scaffold [Mbp]	88.218	87.189	51.802	89.049
Total length [Mbp]	167.447	167.045	173.339	172.603
GC (%)	49	49	49	49
N50 scaffolds [Mbp]	88.218	87.189	37.976	89.049
Number of N’s per 100 kbp	130	76	5,679	999
Number of Gaps	60	23	2,660	144
Complete BUSCOs	995 (98.2%)	986 (97.3%)	990 (97.7%)	989 (96.7%)
Complete and single-copy BUSCOs	985 (97.2%)	976(96.3%)	981 (96.8%)	980 (96.7%)
Complete and duplicated BUSCOs (D)	10 (1.0%)	10 (1%)	9 (0.9%)	9 (0.9%)
Fragmented BUSCOs (F)	6 (0.6%)	7 (0.7%)	9 (0.9%)	10 (1.0%)
Missing BUSCOs (M)	12 (1.2%)	20 (2.0%)	14 (1.4%)	14 (1.4%)
Sequencing platforms	Illumina and PacBio	Illumina and PacBio	Illumina only	Illumina, Hi-C, Nanopore and Optical mapping

**Fig. 2:**
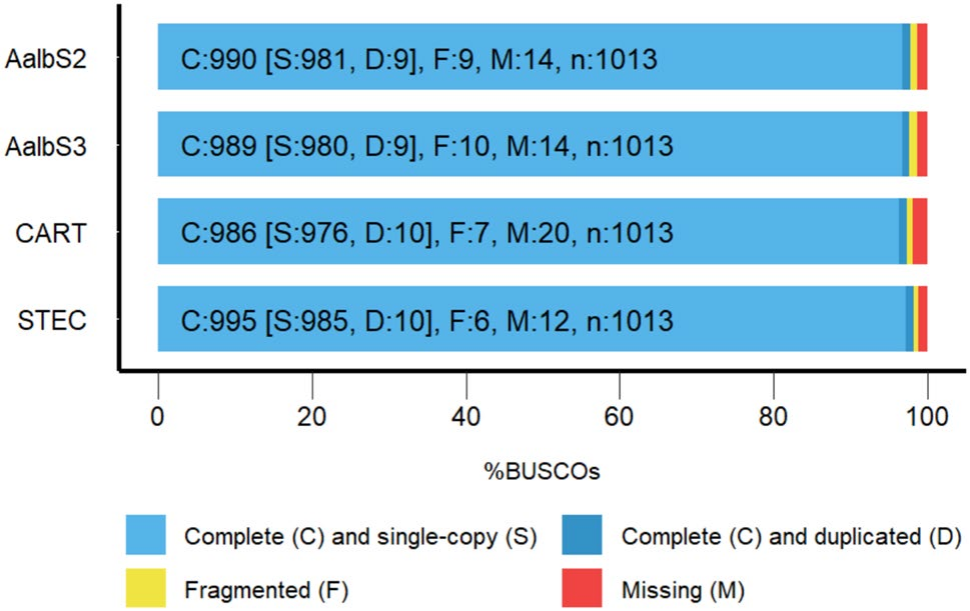
BUSCO assessment results of the assembly completeness of STEC and CART strains as compared with two previously published genome assemblies of An. albimanus (AalbS2 and AalbS3). The completeness of all the assemblies was assessed against the same database of 1013 BUSCO genes from the phylum of Arthropoda.

To assess the genome completeness of the STEC and CART assemblies, we searched each for a set of 1013 ‘benchmark universal single copy orthologs’ (BUSCO) from the Arthropoda class. BUSCO analysis identified 98.2% (995/1013) and 97.3% (986/1013) of the genes in STEC and CART, respectively. Our BUSCO analysis revealed that the completeness of the final assembly of STEC was relatively higher than the completeness of AlbS2 (97.7%) AlbS3 (96.7%) when using the same BUSCO reference database (Table 1, Fig. 2). This supports the high quality of the genome assembly reported in this present sturdy. Additionally, 0.6% and 0.7% of the assessed genes were fragmented, while 1.2% and 2% of them were missing or undetected in STEC and CART respectively (Table 1, Fig. 2). This small percentage of undetected genes may not have significant statistical matches, or the BUSCO matches were scored below the range of scores for the BUSCO profile. Furthermore, undetected genes from the BUSCO analysis may be associated with biological reasons like gene loss or technical reasons such as DNA library preparation artifacts or assembly problems that cannot be solved with the hybrid assembly approach and would require additional sequencing, PCR, and manual analysis^46^. Also, missing sequences in genome assemblies have been reported to be biased toward higher GC and repeat content^63^. We did not investigate the reason for the missing genes in our assemblies. However, based on the consistency in the number of missing genes between the four assemblies assessed, we can speculate that some marker genes included in the BUSCO ‘Arthropoda’ profile used as a reference in this analysis may not be part of the two strains of *An. albimanus* genomes analyzed. Taken together, the generated assemblies have very high contiguity, accuracy, better gene prediction and completeness compared to the previously published genomes of *An. albimanus*.

It is important to highlight that the genome assemblies generated in this study were observed to be more fragmented than the recent hybrid assemblies generated for *An. albimanus* (AlbS3)^23^. This difference in contiguity was expected as the AalbS3 assembly used a combination of four technical approaches including long-read sequencing (Oxford Nanopore), Illumina, Hi-C, and optical mapping. However, this difference in genome contiguity does not invalidate the quality of the assemblies presented here, as several assessment measurements showed their high accuracy and completeness, which make them very useful for further comparative genomics and transcriptomics analyses of *An. albimanus*.

The quality levels of both assemblies were also assessed by mapping the Illumina short reads (DNA-Seq) back to the assemblies. The percentage of reads that was mapped to the final genome in both strains (STEC and CART) were similar to the alignment rate in AalbS3 (~ 92% mapped and ~ 90% properly paired) and relatively higher than the AalbS2 reference genome (87%), validating the de novo assembly and reference-based chromosome scaffolding process (**Figure S2B, Table S5**). Finally, previously published RNA-Seq reads of *An. albimanus* were also mapped to new assemblies and the alignment statistics were compared to AalbS2 and AalbS3. The number of RNA-Seq reads that could be mapped was substantially higher for the new genome assemblies (98.44% for STEC and 86.23% for CART) compared with the older genome assemblies (71.26% for AalbS2 and 71.04% for AalbS3) (**Figure S2 A**, **Table S4**), indicating that the new assemblies may cover more transcriptionally active regions of the genome than the previously published assemblies.

### Gene prediction and functional annotation

A total of 12,120 complete protein coding genes and 13,431 transcript isoforms with an average of 5 exons per gene annotated for the CART assembly, while a total of 12,082 protein coding genes and 13,416 transcript isoforms with an average of 5 exons per gene were identified for the STEC assembly (Table 2**).** An additional 13 protein coding genes were predicted from each mitochondrial genome using MitoZ^48^. While this is substantially higher than the number of protein coding genes detected in AlbS3^23^ and slightly lower than the number in AlabS2^24^, further gene orthogroup and functional analysis including the gene sets of all these assemblies and closest related species are suggested to better understand the reasons behind the differences observed. The gene prediction statistics for both strains are presented in Table 2.

**Table 2:** Summary statistics of the gene prediction and annotation of STEC and CART strains.

Features	CART	STEC
No. of Coding Sequences (CDS)	60,662	61,125
Number of exons	60,637	61,102
Number of protein-coding genes	12,120	12,082
Number of introns	47,232	47,710
Number of start codons	13,431	13,416
Number of stop codons	13,431	13,416
Number of transcripts	13,431	13,416
Total length CDS [kbp] (% of the genome)	23.44 (14.03%)	23.51 (14.04%)
Total length exon [kbp] (% of the genome)	23.41 (14.01%)	23.5 (14.02%)
Total length gene [kbp] (% of the genome)	72.21 (43.23%)	73.6 (43.96%)
Total length intron [kbp] (% of the genome)	53.32 (31.92%)	54.64(32.64%)
Total length transcript [kbp] (% of the genome)	76.75 (45.95%)	78.5 (46.67%)
Transcripts with functional description (%)	10,064 (74.93%)	10, 094 (75.24%)

**Fig. 3:**
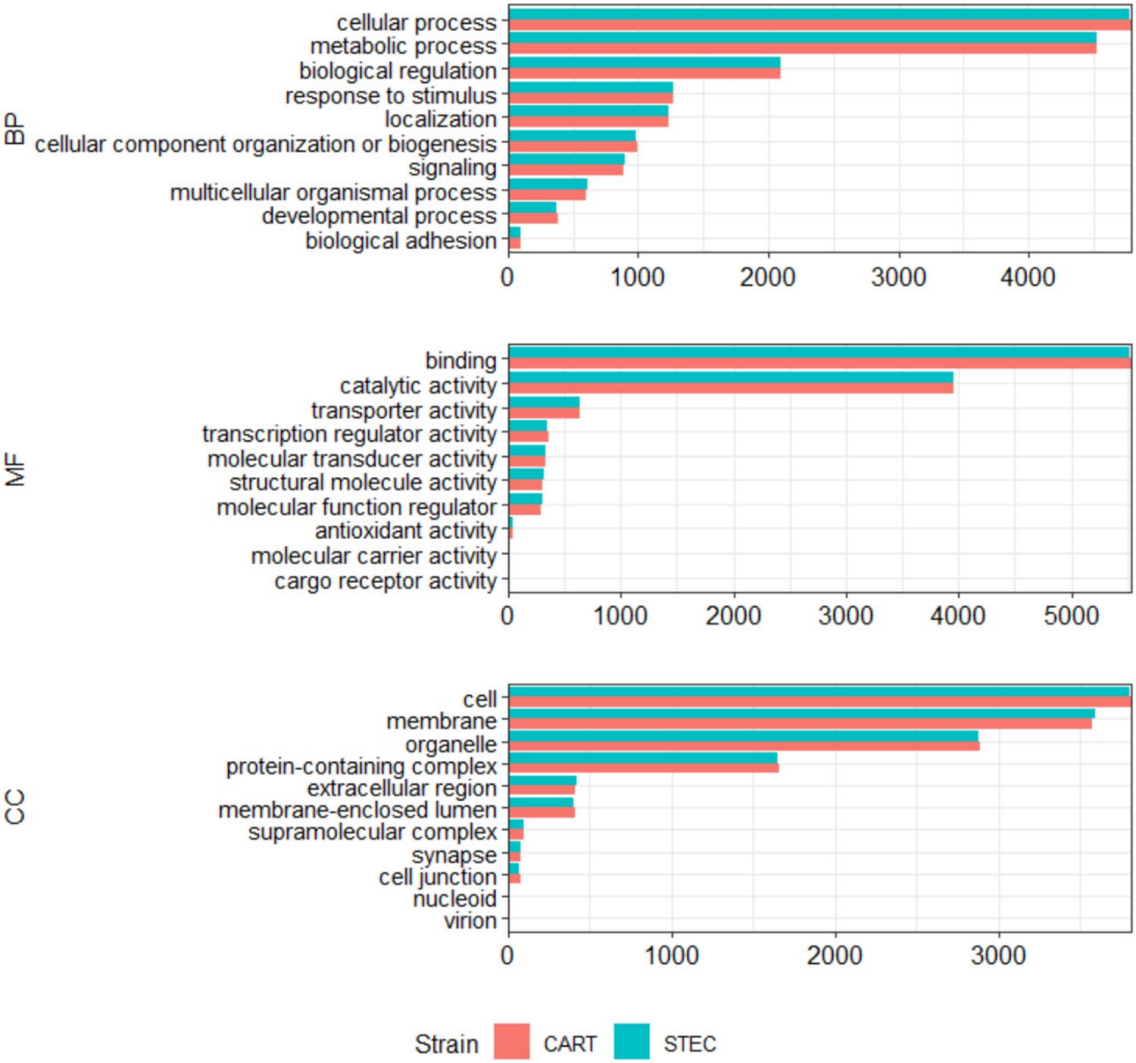
Gene ontology annotation of the predicted transcripts. The bar plots show the number of transcripts assigned the top 10 GO terms for the GO categories BP (biological process), MF (molecular functions), and CC (cellular components) in STEC and CART.

**File S1** and **File S2** describe the assignment of the predicted transcripts to the gene ontology (GO) categories of biological process (BP), molecular function (MF), and cellular component, while the top 10 GO terms are summarized in Fig. 3**.** Importantly, the GO annotation of the predicted transcripts showed similar results for both STEC and CART, indicating the that the two strains have the same metabolic capacity, while top dominant GO terms for the BP categories were ‘cellular process’, ‘metabolic process’, ‘biological regulation’ and ‘response to stimulus.’ The MF gene ontology terms included ‘binding’, ‘catalytic’, ‘transporter’, ‘transcription regulator’, and ‘molecular transducer’ activities (Fig. 3).

### Genomic rearrangements and genetic divergence between the two strains

To assess the structural similarity between two strains, we conducted whole genome alignment of the two assemblies using NUCmer^40^. We found that STEC and CART showed a high degree of similarity (98.12% on average), which is higher than the level of similarity found between the *An. gambiae* and *An. coluzzi* genomes (96.6%), by using the same alignment approach, suggesting that STEC and CART are the same species. To assess whether reference-assisted chromosome scaffolding affected the integrity and uniqueness of each assembly, we used MUMmer to align pre-scaffolded contigs (de novo assemblies) of CART and STEC against AalbS3, as well as CART against STEC. Interestingly, the de novo assemblies of CART and STEC shared 98.00% pairwise identity, while both exhibited 97.76% identity with AalbS3. These values are consistent with the similarity observed in the chromosome-scaffolded genomes, indicating that the chromosome scaffolding approach did not affect the unique structural characteristics of the assemblies.

No major chromosomal rearrangements were detected between STEC and CART. Out of a total of 149 assembled scaffolds in CART, 115 matched 91 of the 109 assembled scaffolds in STEC. A total of 19 and 35 scaffolds, representing 0.05% and 0.1% of the genome size in STEC and CART, respectively, did not align and suggest the presence of strain-specific DNA. Interestingly, no protein coding genes were predicted from most of the strain-specific scaffolds. From the STEC-specific scaffolds, only two protein-coding genes were predicted, while from CART-specific scaffolds, thirteen protein-coding genes were predicted. The functional annotation of the few genes predicted from species-specific scaffolds is reported in **Table S6**. Taken together, the strain-specific scaffolds, which represent a minor fraction of the genome size, mostly comprised of non-coding and low-complexity DNA, suggesting that they will have little effect on the gene content and metabolic capacity of the two strains. Furthermore, comparisons of 11,221 gene pairs revealed no genomic rearrangements in the STEC or CART strain (**Figure S3**), indicating that the possibility of cryptic speciation in *An. Albimanus* is unlikely, in contrast to *An. gambiae* complex that is known to have multiple cryptic species^64^.

### Analysis of single nucleotide polymorphisms and indels

Based on our BUSCO analysis, the assembly of the STEC strain presented here is considered the most comprehensive and complete assembly for *An. albimanus* (Table 1, Fig. 1). We compared the Illumina sequences of both the STEC and CART genomes using a SNP calling approach, by considering the final assembly of STEC as the reference. The results of this analysis are summarized in **Table S7**. A total of 1,550,575 and 563,725 SNPs, and 271,926 and 98,485 indels were detected in whole genome sequences of CART and STEC, respectively. However, using SnpEff^57^, we imputed that the overall functional impact of all SNPs were mostly modifiers (~ 95.9%), followed by low (~ 2.8% %) moderate (~ 1.3%) and high impact (~ 0.02%); while a larger fraction of the SNPs detected (~ 68%) were silent and had no impact on the gene functions **(Figure S4**, **Table S7**). The negligible fraction of variants detected to have high functional effects on the coding regions affected 123 and 331 protein coding genes in STEC and CART, respectively. These genes were not significantly over- or underrepresented in any biological process, supporting the finding of our functional annotation, which suggested that the STEC and CART strains did not differ in metabolic characteristics. Using the same SNP calling pipeline, the number of SNPs identified using STEC as reference for both strains is consistent with the results found by using AlbS3 as a reference. This is not surprising since AlbS3 was also assembled from the *An. albimanus* Stecla strain originating from El Salvador^23^. Thus, the large difference observed in the number of SNPs and indels between the two strains may be related to their different geographical origins. While this SNP analysis suggests some genetic differences between the two populations, this is not sufficient to infer population genomic structures of the original field populations, since they were colonized for several years at the Malaria Research and Reference Reagent Resource (MR4) at US Centers for Diseases Control and Prevention (CDC) and the Instituto Nacional de Salud in Bogota, Colombia. Thus, a large fraction of the mutations observed may be attributed to genetic drift associated with the resulting reduction in effective population size.

### Mitochondrial genomes

**Fig. 4:**
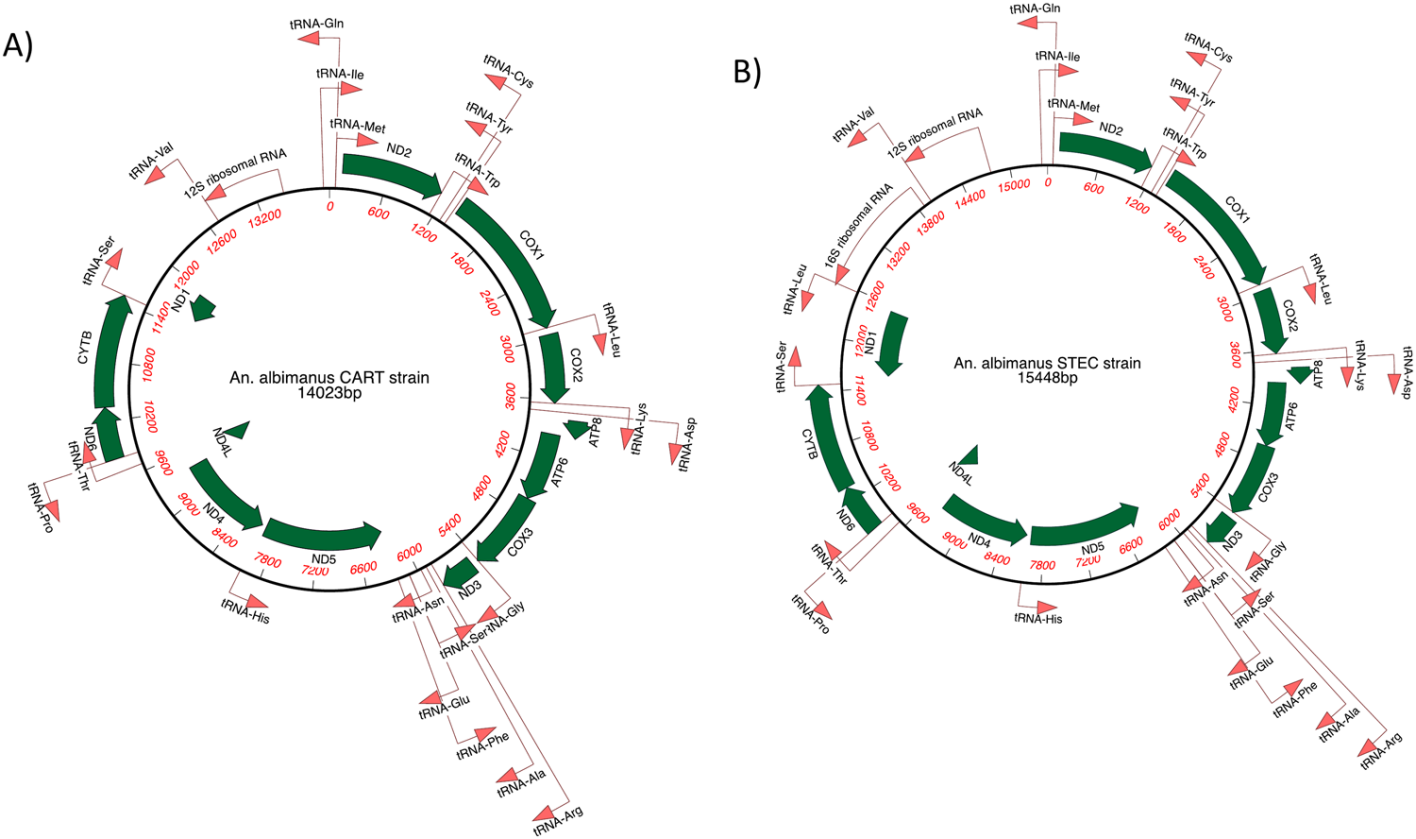
The organization of the mitochondrial (mt) genomes of *An. albimanus* CART strain (**A**) and STEC strain (**B**). All the CDS corresponding to each coding gene are shown in dark green, while the tRNAs and rRNAs are represented with a red line arrow. The direction of the arrows for each gene represents the transcription direction. Protein coding genes are represented using standard nomenclature, while tRNAs are represented using three letter amino acid codes. In total, 13 protein coding genes were identified from the mt genomes of each strain. Gene length scaling is approximate.

The complete mitochondrial (mt) genomes of both STEC and CART were generated from our hybrid assembly approach. Our results indicated that the complete mt genomes of STEC and CART consisted of 15,448 and 14,023 nucleotides, respectively, and had a GC content of ~ 23%. The nucleotide composition was similar for the two strains STEC (A = 40%, T = 37%, G = 10%, C = 13%) and CART (A = 40%, T = 38%, G = 10%, C = 13%). The NUcmer alignment of the two mt genomes revealed that they shared 99.96% pairwise identity. The gene prediction of the mt genomes was conducted using MitoZ and a total of 37 genes (2 rRNAs, 22 tRNAs, 13 protein coding genes) and 35 genes (1 rRNA, 21 tRNAs, 13 protein coding genes) were detected in the mt genomes of STEC and CART, respectively. We observed that two genes including l rRNA and 1 tRNA (tRNA-leu) were missing from the annotation of the CART mt genome, suggesting that the annotation of the STEC mt genome was more complete. The mt genes are highly conserved across all Culicidae^65^, so the absence of two genes in the CART mt genome is more likely the result of misassembly rather than true gene loss. Importantly, the two mt genomes shared all of the recognizable protein encoding genes (ATP6, ATP8, COX1, COX2, COX3, CYTB, ND1, ND2, ND3, ND4, ND4L, ND5, ND6) and 21 tRNAs representing 20 amino acids and 1 rRNA (s-rRNA) (Fig. 4). Interestingly, the key protein coding genes identified here are consistent with other *Anopheles* mt genomes previously annotated^66–68^, evidencing good quality of the assembled mt genomes.

Taken together, the mt genomes of both strains were relatively similar in size, GC content, nucleotide composition (99.6% similarity) and protein coding genes. Previous studies have shown that intraspecific variation in complete mitochondrial genomes of *Anopheles* species is low, typically less than 2%^69,70^. While individual mitochondrial genomes may not reliably distinguish cryptic species due to potential introgression events^65^, this high level of similarity of both mitochondrial and nuclear genomes of CART and STEC is consistent with observations in other Anopheles species and suggest that these strains belong to the same species.

### Transposable element analysis

**Fig. 5:**
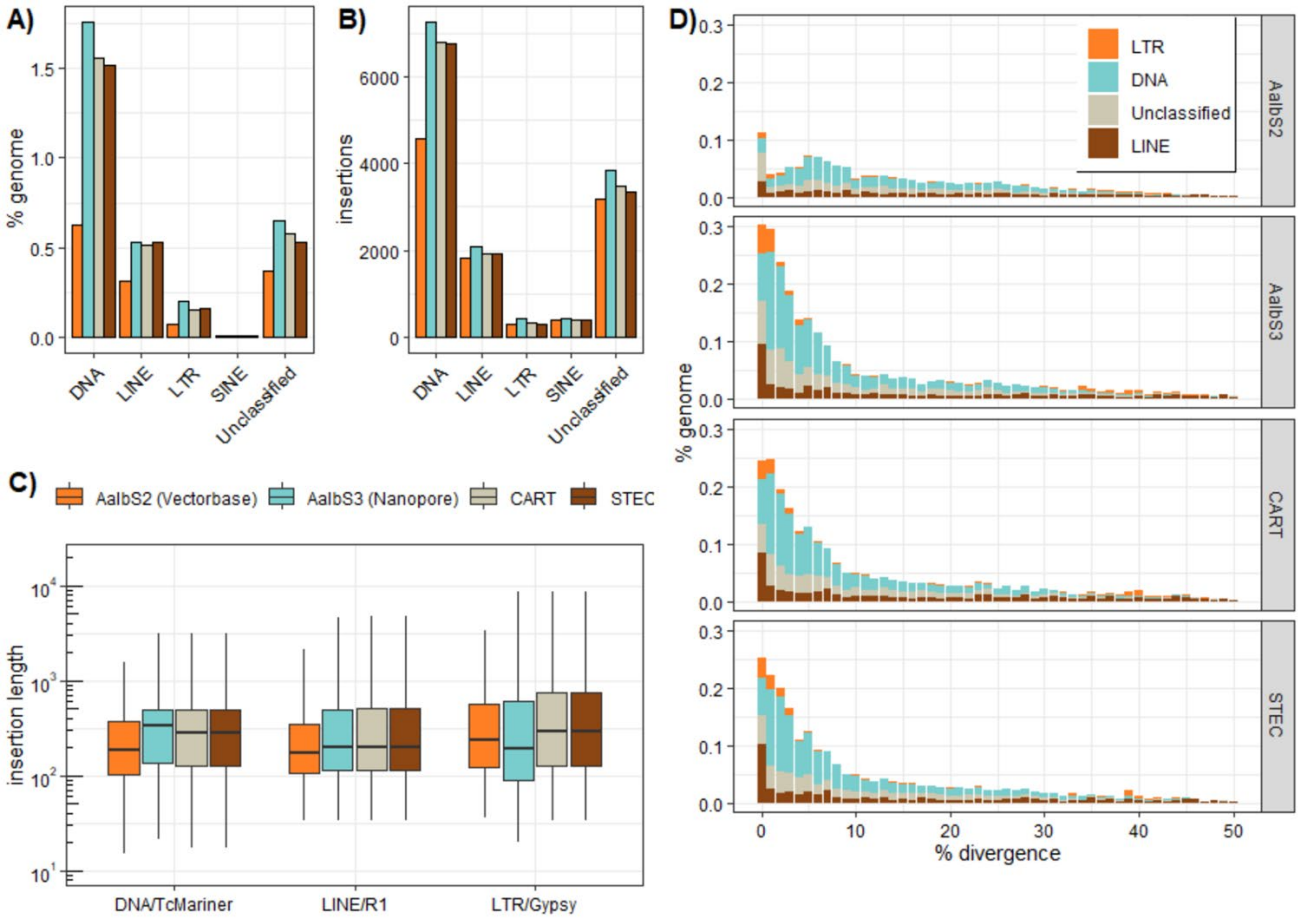
Transposable element (TE) analyses, showing (**A***)* Relative proportions (%) of DNA transposons, LTR, LINE and SINE (short interspersed nuclear elements) retrotransposons identified in the genome assembly generated by this study (STEC and CART), AalbS2 (Vector base) and AalbS3 (NCBI); (**B**) Total amount of genomic insertion associated with different TE families; (**C***)* The insertion length distribution. A) Percentage of each genome comprised of the major TE types estimated by RepeatMasker. B) Number of insertions of each TE types discovered in each draft. C) Length distribution of insertions from the longest DNA transposon, LINE, and LTR family. D) Accumulation history of TE types derived from measuring the K2P divergence between each TE insertion and the consensus element. Lower divergences indicate TEs inserted more recently whereas older insertions have higher divergences.

We performed a repeat analysis of the PacBio assemblies to test whether more TE content was recovered as compared to AalbS3. We identified 115 TE families; of these, 20 LINEs, 8 LTRs, 51 DNA, 1 SINE, and 35 were unclassified. We masked all *An. albimanus* genomes with RepeatMasker and filtered redundant repeats following the methodology described by Vandewege et al.^71^. Overall, TE content was low and 2.8%, 2.9%, and 3.2% of the STEC, CART and AalbS3 drafts were repetitive, respectively, but the repetitive content of AalbS2 was only 1.4% (Fig. 5A). Therefore, the hybrid genome assemblies captured twice as much repetitive content compared to as the short-read-only assembly. The most common TEs were DNA transposons, followed by unclassified elements, LINEs, LTRs, and SINEs (Fig. 5A). The hybrid genome assemblies captured more TE content in terms of percent of the assembly (Fig. 5A) and number of insertions (Fig. 5B). For long representatives (i.e. TcMariner DNA transposons, R1 LINEs, and Gypsy LTRs), insertions were longer in the long-read assemblies, however, the median LTR Gypsy length was lowest in the AalbS3 draft (Fig. 5C).

To estimate the mobilization history of TEs in *An. albimanus*, we calculated the K2P distance between each insertion and the consensus element representing the master mobilizing element. We could infer that the mobilizing elements were reasonably young, as most insertions had a K2P divergence of less than 10% (Fig. 5D) and older elements were purged from the genome relatively quickly. We were also able to determine that the short read assembly was largely lacking this young repetitive content, likely because TEs were so similar that reads derived from those TEs collapsed together which underestimated the repeat content. Importantly, the amount or percentage of TE content was positively correlated with the genome size of the three hybrid assemblies. This indicates that the slightly higher genome size of AalbS3 compared to STEC and CART could arise primarily through the accumulation of TEs, which is consistent with previous findings^72^.

## Conclusions

We obtained high-quality genome assemblies of two geographically distinct strains of *An. albimanus* by combining long-read sequences generated by PacBio sequencing and short-read sequences generated by Illumina sequencing. The STEC and CART strains, were originally colonized from El Salvador and Colombia, respectively. Our hybrid assembly approach generated high quality genomes for each strain and recovered ~ 96% of the expected genome size (173 Mbp). The genome assemblies of STEC and CART consisted of 109 and 149 scaffolds, respectively, with estimated genome sizes of 167.5 Mbp (N_50_ = 88 Mbp) and 167.1 Mbp (N_50_ = 87 Mbp), respectively. The resulting genome assemblies for each strain were organized in three chromosomes, complete mitochondrial genomes, and several unplaced scaffolds. We demonstrated significant improvement in the completeness, accuracy, and contiguity of the assemblies compared to non-hybrid and hybrid assemblies available for *An. albimanus*. Although the strains were colonized from two geographically distinct populations, the alignment of the two exhibited a high level of genomic similarity. In addition, comparisons of orthologous gene pairs revealed no major genomic rearrangements in STEC and CART, suggesting that the two strains belong to the same species. However, we found evidence of strain-specific DNA and mutations, highlighting some differences between the two strains. To date, these are the first *An. albimanus* genomes co-assembled with high coverage Illumina short-reads and PacBio long reads. As such, these assemblies provide a useful resource for comparative genomics, proteomics, epigenetics, transcriptomics, and functional analyses of this important malaria vector.

## Supplementary Information

Supplementary Information 1.

Supplementary Information 2.

Supplementary Information 3.

## Data Availability

The raw sequencing reads generated for this study (PacBio and Illumina), the final polished assemblies and annotation were deposited in NCBI under project accession PRJNA803167. The protein-coding genes, Gene Annotation Format (GTF) and the final assembly files were also deposited at the open science framework https://osf.io/4n7vh/files/osfstorage (user account required).

## Code availability

Custom scripts used for all the analyses are available from the authors on request.
