# Supplementary material for: Chromosome-scale genome assembly and annotation of two geographically distinct strains of malaria vector Anopheles albimanus
Source: Sci Rep. 2025 Jun 3;15:19448. doi: 10.1038/s41598-025-01713-9 (PMC12134381; doi:10.1038/s41598-025-01713-9)
Supplement: Supplementary file 3 — Supplementary Information 3. [file 41598_2025_1713_MOESM3_ESM.pdf]

Chromosome-Scale Genome Assembly and Annotation of Two Geographically Distinct Strains of Malaria Vector *Anopheles albimanus*

Dieunel Derilus[1*], Gareth D. Weedall[2], Michael W. Vandewege[3], Dhwani Batra[4], Mili Sheth[5], Lori A. Rowe[6], Ananias A. Escalante[7], Audrey Lenhart[1], Lucy Mackenzie Impoinvil[1*]

[1]Entomology Branch, Division of Parasitic Diseases and Malaria, National Center for Emerging and Zoonotic Infectious Diseases, Centers for Disease Control and Prevention, Atlanta, Georgia, USA

[2]School of Biological and Environmental Sciences, Liverpool John Moores University, Liverpool, UK

[3]Department of Clinical Sciences, College of Veterinary Medicine, North Carolina State University, Raleigh NC 27607

[4]Office of Advanced Molecular Detection, Division of Infectious Disease Readiness and Innovation, Centers for Disease Control and Prevention, Atlanta, Georgia, USA.
[5]Biotechnology Core Facility Branch, Division of Core Laboratory Services and Response. Office of Laboratory Safety and Response. Centers for Disease Control and Prevention, Atlanta, Georgia, USA.
[6]Tulane National Primate Research Center, Department of Microbiology, Viral Characterization, Isolation, Production and Sequencing Core, Tulane University, Covington, Louisiana, USA

[7]Department of Biology/Institute for Genomics and Evolutionary Medicine, Temple University, Philadelphia, Pennsylvania, USA.

Correspondence to D.D(qlk5@cdc.gov) and L.M.I (ykd8@cdc.gov)

Supplementary Materials

Supplementary Figures

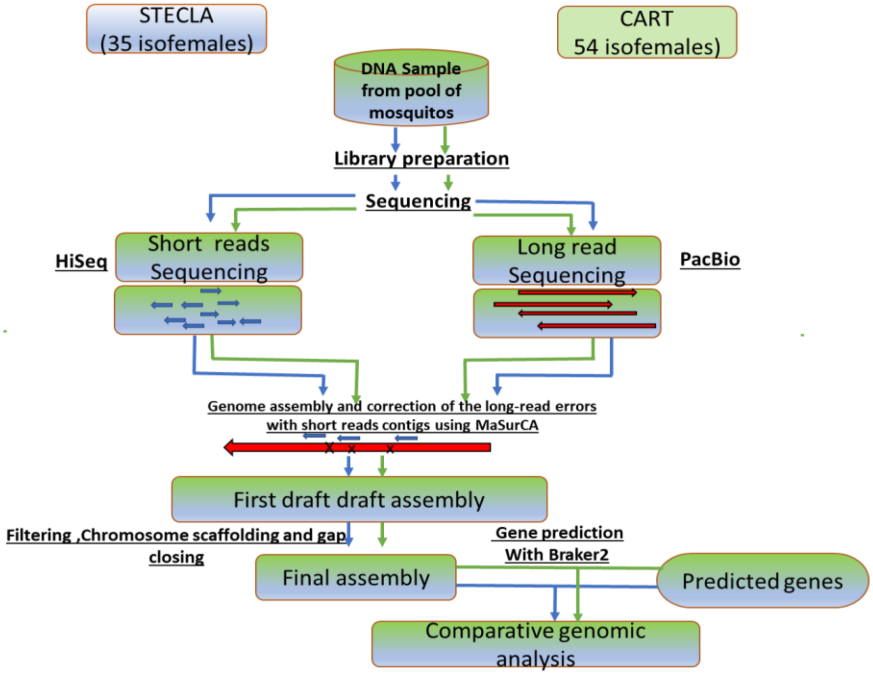

Figure S1: Flowchart of the sample processing and hybrid assembly pipeline used to integrate the Illumina short reads and the PacBio long reads. The hybrid genome assembly was conducted using MaSurCA software

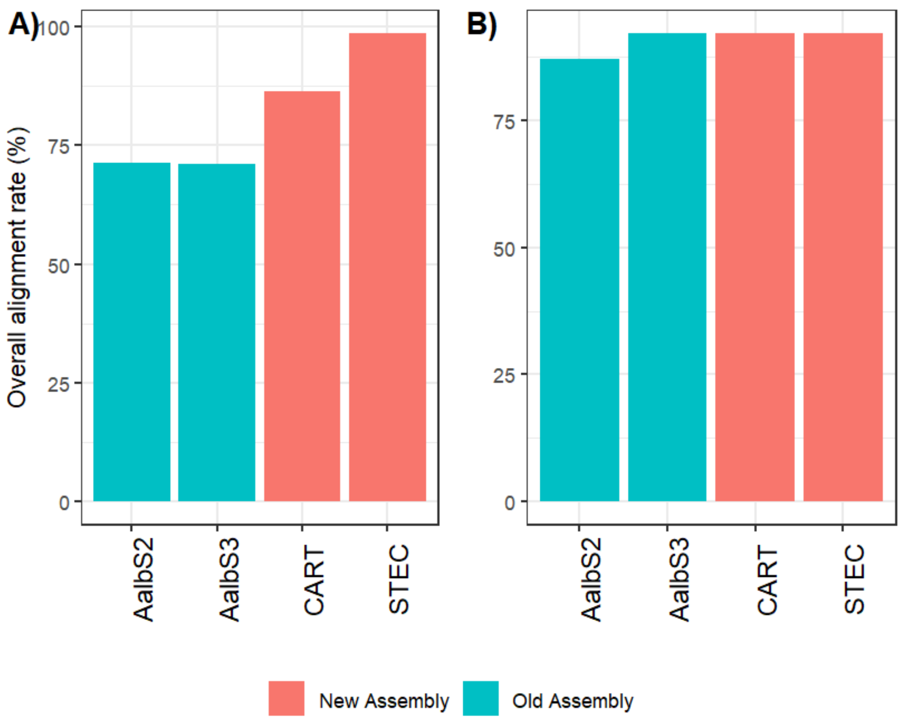

Figure S2: Comparison of mapping results of RNA-seq and DNA-seq paired-end reads onto the new (from this study) and the previous genome assemblies. A) RNA-seq alignment results; B) DNA-Seq alignment results.

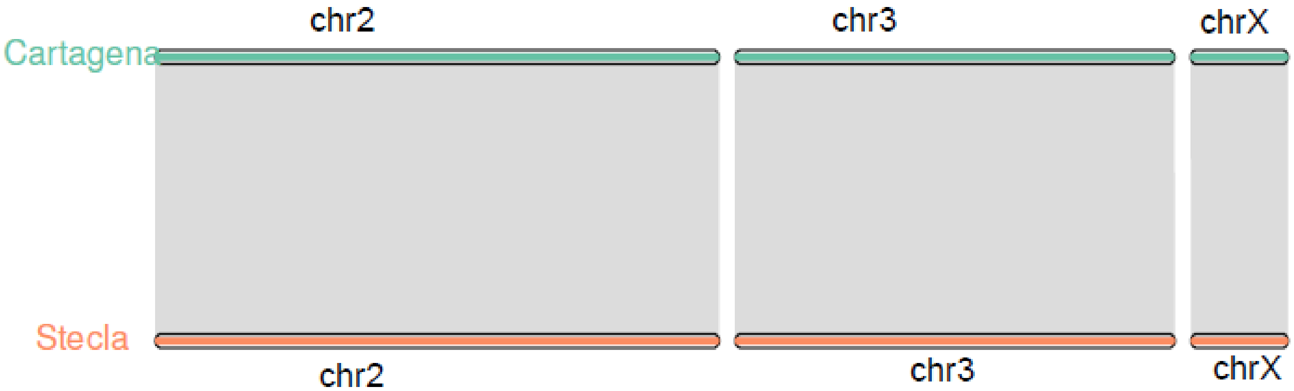

Figure S3: Synteny relationship between CART and STEC assemblies. The genome synteny analysis was performed with 11,221 orthologous gene pairs between the two species.

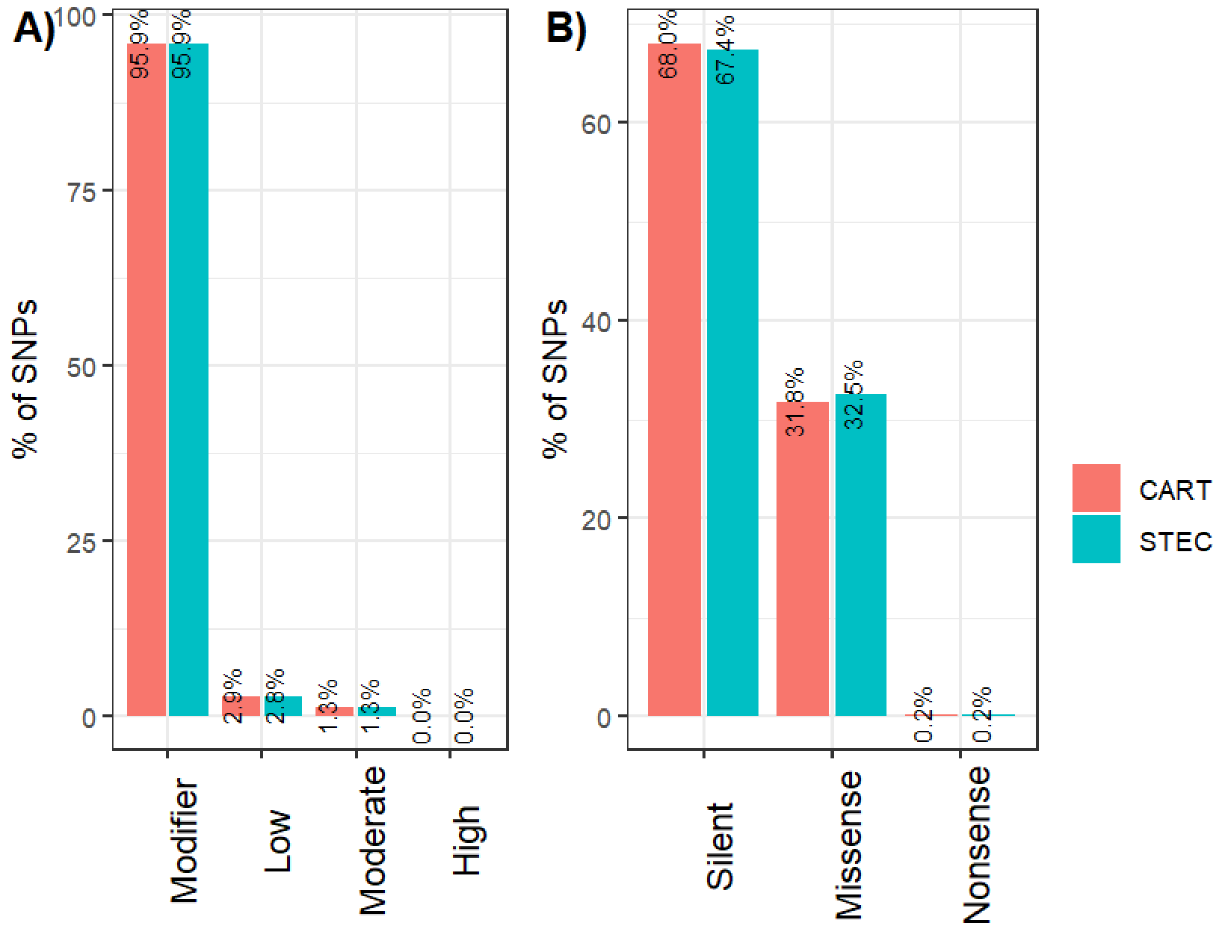

Figure S4: Results Statistics of the SNP annotation A) Number of effects by impact; B) Number of effects by functional class. A negligible fraction of the detected SNPs was observed to have high impact on the protein coding genes. SNP annotation was performed using SnpEff.

Supplementary tables

Table S1: The analysis report for the PacBio reads generated by Nanostat.

	STEC	CART
**General summary:**		
Mean read length:	5,136.80	6,891.60
Mean read quality:	8.3	8.3
Median read length:	4,652.00	6,027.00
Median read quality:	8.5	8.5
Number of reads:	1,675,245.00	1,444,111.00
Read length N50:	6,826.00	9,896.00
STDEV read length:	3,807.10	5,028.90
Total bases:	8,605,378,183.00	9,952,264,557.00
**Number, percentage and megabases of reads above quality cutoffs**		
>Q5:	1654461 (98.8%) 8542.5Mb	1427570 (98.9%) 9907.3Mb
>Q7:	1440185 (86.0%) 7383.0Mb	1261097 (87.3%) 8837.7Mb
>Q10:	6513 (0.4%) 11.9Mb	4795 (0.3%) 11.3Mb
>Q12:	38 (0.0%) 0.0Mb	22 (0.0%) 0.0Mb
>Q15:	0 (0.0%) 0.0Mb	0 (0.0%) 0.0Mb
**Top 5 highest mean basecall quality scores and their read lengths**		
1:00	14.0 (1)	14.0 (1)
2:00	14.0 (2)	14.0 (1)
3:00	14.0 (1)	13.5 (9)
4:00	13.5 (2)	13.1 (8)
5:00	13.4 (5)	13.0 (8)
**Top 5 longest reads and their mean basecall quality score**		
1:00	60081 (8.2)	62809 (8.0)
2:00	58100 (5.2)	60697 (9.0)
3:00	55565 (4.7)	59522 (6.8)
4:00	54648 (7.3)	58586 (6.9)
5:00	53788 (7.3)	58312 (7.1)

Table S2: Summary statistics generated with 'quast' comparing the performance of the de novo assemblies (before chromosome scaffolding) produced by the hybrid (using MaSuRCA) and the non-hybrid approaches.

	CART		STECLA	
Assembly	**Flye (non-hybrid)**	**MaSuRCA (hybrid)**	**Flye (non-hybrid)**	**MaSuRCA (hybrid)**
# contigs (>= 0 bp)	312	227	520	322
# contigs (>= 1000 bp)	266	203	454	299
# contigs (>= 5000 bp)	145	145	306	254
# contigs (>= 10000 bp)	96	114	235	227
# contigs (>= 25000 bp)	66	77	155	178
# contigs (>= 50000 bp)	45	64	137	165
Total length (>= 0 bp)	167,844,140	166,819,213	168,634,144	167,193,322
Total length (>= 1000 bp)	167,812,774	166,803,677	168,586,854	167,177,487
Total length (>= 5000 bp)	167,485,736	166,643,310	168,196,670	167,058,506
Total length (>= 10000 bp)	167,147,489	166,429,764	167,695,251	166,853,766
Total length (>= 25000 bp)	166,704,223	165,767,940	166,478,160	165,999,220
Total length (>= 50000 bp)	166,042,922	165,300,642	165,874,963	165,575,236
# contigs	312	227	520	322
Largest contig	27,967,288	26,134,696	4,976,363	7,705,473
Total length	167,844,140	166,819,213	168,634,144	167,193,322
GC (%)	49	49	49	49
N50	17,497,647	5,466,899	2,181,744	1,995,104
N75	5,360,629	3,318,873	1,200,190	941,277
L50	4	7	24	23
L75	8	17	51	54
# N's per 100 kbp	1	1	2	1
Gaps	18	17	32	15

Table S3: Summary statistics generated with 'quast' comparing the performance of the de novo assemblies (after chromosome scaffolding) produced by the hybrid (using MaSuRCA) and the non-hybrid approaches.

	**CART**		**STECLA**	
	**Flye (non-hybrid)**	**MaSuRCA (hybrid)**	**Flye (non-hybrid)**	**MaSuRCA (hybrid)**
# contigs (>= 0 bp)	184	**149**	267	**109**
# contigs (>= 1000 bp)	184	149	267	109
# contigs (>= 5000 bp)	80	89	134	68
# contigs (>= 10000 bp)	43	56	70	47
# contigs (>= 25000 bp)	21	18	13	14
# contigs (>= 50000 bp)	10	8	5	5
Total length (>= 0 bp)	168,295,510	167,045,586	169,257,486	167,447,072
Total length (>= 1000 bp)	168,295,510	167,045,586	169,257,486	167,447,072
Total length (>= 5000 bp)	168,007,681	166,886,184	168,909,055	167,340,004
Total length (>= 10000 bp)	167,752,983	166,661,627	168,451,026	167,178,357
Total length (>= 25000 bp)	167,426,749	165,981,630	167,640,911	166,603,563
Total length (>= 50000 bp)	167,047,972	165,596,376	167,359,165	166,290,968
# contigs	184	149	267	**109**
Largest contig	88,256,638	87,189,253	88,657,803	88,217,557
Total length	168,295,510	167,045,586	169,257,486	167,447,072
GC (%)	49	49	49	49
N50	88,256,638	87,189,253	88,657,803	88,217,557
N75	65,959,180	65,752,556	66,252,363	65,929,290
L50	1	1	1	1
L75	2	2	2	2
# N's per 100 kbp	145	**76**	256	**130**
Gaps	43	**23**	127	**60**

Table S4: Comparison of the alignment results of RNA-Seq reads (generated from *An. albimanus*) against the polished assemblies generated in this study (STEC and CART) and previously published reference genomes (AalbS2 and AalbS3).

Reference genome	Reads to align (R1+R2)	Aligned reads (%)[1]	Properly paired (%)[2]	Aligned reads, filtered (%)[3]
AalbS2	51,437,712	36657028(71.26%)	35926252(69.84%)	35899012 (97.93%)
AalbS3	51,437,712	36539861(71.04%)	35868104(69.73%)	35724080 (97.77%)
Cartagena	51,437,712	44356729(86.23%)	43689686(84.94%)	43731327 (98.59%)
Stecla	51,437,712	50635363(98.44%)	50014842(97.23%)	50318087 (99.37%)

[1] % RNA-seq reads mapped to the reference genome

[2] Both the read and its mate (forward and reverse) are aligned to the opposing strands of the reference genome

[3] % of filtered aligned reads (q>10)

Table S5: Alignment results of Illumina Hi-Seq paired end reads from against the polished genome assembly STECLA.

Population	Reference genome	Read to align (R1+R2)	Aligned reads (%)[1]	Properly paired (%)[2]	Aligned reads, filtered (%)[3]
CART	STEC	403,726,572	373,265,142 (92.45 %)	361,492,574(96.85 %)	363,011,160 (97.25 %)
STEC	STEC	462,584,846	424,455,286 (91.76 %)	415,891,546(97.98 %)	413,226,758 (97.35 %)
CART	CART	403,726,572	376,551,859 (93.27 %)	366,485,414(97.33 %)	364,757,037 (96.87 %)
STEC	CART	462,584,846	424,066,148 (91.67 %)	415,251,334(97.92 %)	410,696,946 (96.85 %)
CART	AlbS3	403,726,572	374,079,879 (92.66 %)	364,464,886(97.43 %)	344,947,233 (92.21 %)
STEC	AlbS3	462,584,846	423,495,968 (91.55 %)	364,464,886(86.06 %)	400,130,133 (94.48 %)
CART	AlbS2	403,726,572	356,460,556 (88.29 %)	338,866,164(95.06 %)	353,328,681 (99.12 %)
STEC	AlbS2	462,584,846	409,562,390 (88.54 %)	395,039,988(96.45 %)	338,108,980 (82.55 %)

[1] % RNA-seq reads mapped to the reference genome

[2] Both the read and its mate (forward and reverse) are aligned to the opposing strands of the reference genome

[3] % of filtered aligned reads (q>10)

Table S6: Blast2GO annotation of the genes predicted from the scaffolds that did not align between the two assemblies.

Gene ID	Scaffold	Description	GO Names
**CART**			
CART_012049	unplaced_scaffold_11	F-box LRR-repeat 6	F: protein binding
CART_012050	unplaced_scaffold_11	chondroitin proteoglycan 2-like	F: chitin binding; C:extracellular region
CART_012051	unplaced_scaffold_11	obstructor-E-like isoform X2	F: chitin binding; C:extracellular region
CART_012052	unplaced_scaffold_11	probable chitinase 10	F: chitin binding; C:extracellular region
CART_012053	unplaced_scaffold_11	isoform A	
CART_012054	unplaced_scaffold_11	phd finger	F:DNA binding; F:hydrolase activity; F:metal ion binding; C:nucleus
CART_012056	unplaced_scaffold_118	---NA---	
CART_012061	unplaced_scaffold_121	uncharacterized protein LOC118468338	P:DNA integration; F:nucleic acid binding
CART_012061	unplaced_scaffold_122	uncharacterized protein LOC118468338	P:DNA integration; F:nucleic acid binding
CART_012080	unplaced_scaffold_39	opioid-binding cell adhesion molecule	F:protein binding; C:integral component of membrane
CART_012084	unplaced_scaffold_46	proton-coupled amino acid transporter CG1139	C:integral component of membrane
CART_012085	unplaced_scaffold_46	limbic system-associated membrane	
CART_012099	unplaced_scaffold_76	uncharacterized protein LOC121590437	
**STEC**			
STEC_012044	unplaced_scaffold_17	reverse transcriptase	P:RNA-dependent DNA biosynthetic process; F:RNA-directed DNA polymerase activity
STEC_012068	unplaced_scaffold_86	repetitive proline-rich cell wall -like	

Table S7: Statistics of the SNP annotation results.

	CART	STEC
**Number of Mutations**		
SNPs	1550575	563725
Indels	271,926	98,485
**Predicted SNP effect**		
Missense	32,859 (31.84 %)	11,687 (32.45%)
Nonsense	160 (0.16 %)	53 (0.15%)
Silent	70,170 (68.0 %)	24280 (67.41%)
**SNP impact**		
High	388 (0.02%)	134 (0.02%)
Low	74389 (2.89%)	25662 (2.79%)
Moderate	32751 (1.27%)	11648 (1.26%)
Modifier	2468924 (95.93%)	883963 (95.94%)
**SNP effect by region**		
Donwstream	478756 (18.58%)	168202 (18.26%)
Exon	102585 (3.98%)	35821 (3.89%)
Intergenic	929845 (36.09%)	336481 (36.52%)
Intron	550136 (21.35%)	201453 (21.86%)
Spice_site_acceptor	60 (0.00%)	21 (0.00%)
Splice_site_donor	69 (00%)	24 (0.00%)
Splice_site_region	4814(0.19%)	
Transcript	3987 (0.16%)	1771 (0.19%)
Upstream	506200 (19.65%)	176056 (19.11%)

Supplementary Files

File S1: Blast2 GO annotation results of the predicted transcripts in STEC strain (xlsx)

File S2: Blast2 GO annotation results of the predicted transcripts in CART strain (xlsx)
